# Dutch Preadolescents’ Food Consumption at School: Influence of Autonomy, Competence and Parenting Practices

**DOI:** 10.3390/nu13051505

**Published:** 2021-04-29

**Authors:** Roselinde L. van Nee, Ellen van Kleef, Hans C. M. van Trijp

**Affiliations:** Marketing and Consumer Behavior Group, Wageningen University and Research, 6706 KN Wageningen, The Netherlands; ellen.vankleef@wur.nl (E.v.K.); hans.vantrijp@wur.nl (H.C.M.v.T.)

**Keywords:** healthy eating, adolescents, autonomy, competence, motivation, parenting

## Abstract

Eating habits appear to become less healthy once children move into adolescence. Adolescence is characterized by increasing independence and autonomy. Still, parents continue influencing adolescents’ eating habits. This cross-sectional study used a Self-Determination Theory perspective to examine how parents can support preadolescents’ food-related autonomy and competence and how these factors are associated with healthy eating motivation and food consumption at school. In addition, the effect of relative healthy food availability at home on preadolescents’ food consumption at school was explored. In total, 142 Dutch preadolescents (mean age 12.18) and 81 parents completed questionnaires. The results showed that preadolescents perceived themselves as having higher food-related autonomy and lower competence to eat healthily as compared to their parents’ perceptions. A path analysis was conducted to test the hypothesized model. Although parental support was positively associated with food-related autonomy, higher food-related autonomy was related to less healthy food intake at school. On the other hand, competence to eat healthily indirectly affected preadolescents’ healthy intake ratio through their healthy eating motivation. Finally, the relative availability of healthy options at home was positively associated with preadolescents’ healthy intake ratio outside the home. Findings from the study advance the understanding of individual and environmental factors that influence eating habits during the key life period of early adolescence. The results may inform interventions aiming to guide preadolescents to make healthy food choices on their own.

## 1. Introduction

Healthy eating habits during childhood and adolescence are important for youth development and long-term health. However, eating habits appear to become less healthy from childhood to adolescence. As children move into adolescence, their consumption of unhealthy foods, including sugar-sweetened beverages, has been found to increase, whereas their consumption of healthy foods, including fruits and vegetables, decreases [1,2,3]. This is alarming, as adolescents’ eating habits track into adulthood [4,5]. The deterioration in diet quality may be explained by increasing independence, which characterizes the developmental transition from childhood to adolescence [3]. More specifically, preadolescents (aged 10–14 years) are able to make more food-related decisions than younger children, for example, regarding the timing and location of food consumption [6,7,8]. Particularly the school environment is recognized as an important setting for independent eating occasions, as approximately one-third (35%) of daily food intake is consumed at school [9]. In order to develop effective approaches to promote healthy diets, it is important to understand individual and environmental factors that influence eating habits during this key life period [10].

According to the Self-Determination Theory (SDT), autonomy, competence and supportive parenting practices are important factors to learn and maintain healthy eating habits [11,12]. To make healthy food choices, one needs to feel that one has a choice (e.g., autonomy) and a feeling of personal effectiveness (e.g., have competence) according to the SDT. Together with the need to feel related to significant others, the satisfaction of these psychological needs increases intrinsic motivation to behave in a certain way [13]. Furthermore, the SDT emphasizes the central role of parents in supporting their children’s needs for autonomy and competence [11,12]. Part of the support for preadolescents may be in leading by example, such as in the availability of foods at home [14]. SDT has been widely applied as a theoretical framework for studying influencing factors on eating behavior [15,16]. The majority of studies on eating behavior in the context of SDT have been conducted with adults (e.g., [17] (cross-sectional), [18,19] (longitudinal)) and older adolescents (e.g., [20] (cross-sectional), [21] (longitudinal)). However, early adolescence at 10–14 years of age is a transitional period in life with important developmental, social and environmental changes that may impact behaviors [22]. Therefore, the current study will examine how food-related autonomy, competence and supportive parenting practices are related to healthy eating motivation and food choices in preadolescents.

Early adolescents from 10 to 13 years old frequently engage in independent eating occasions [8]. In the Netherlands, primary school children bring their own morning snack and beverages to school. Schools can have food policies, for example, about the foods and beverages children are allowed to bring [23]. Most schools have a continuous schedule, meaning that children stay at school to consume a home-packed lunch during the break. A typical lunch for Dutch primary school children consists of a sandwich with a sweet or savory filling and a beverage [24]. The morning snack can be anything, such as fruit, biscuits, bread or sweets. Intervention research has tried to improve the healthiness of this snack choice at primary schools [25].

Despite the increased opportunities to make independent food choices, parents seem to continue influencing adolescent eating habits through their parenting practices [26]. It has been suggested that parenting practices are based on parental perceptions regarding adolescents’ food-related autonomy and competence [27]. However, parental perceptions may not correspond to how preadolescents perceive themselves. For example, if preadolescents feel that they are highly competent to make healthy food choices but their parents do not agree, the parents may enforce strict food rules. Consequently, preadolescents may perceive that they have low food-related autonomy, which may result in unhealthy food choices. Previous cross-sectional studies have often reported low agreement between adolescent and parent reports on different parenting and eating behaviors, suggesting that adolescents and parents seem to perceive behaviors differently, e.g., [28,29]. For example, it was found that adolescents’ behavioral skills regarding fruit or vegetables, such as cutting fruits or vegetables, were rated higher among adolescents aged 10–12 than their parents [30]. Another study reported that adolescents perceived that they were more often involved in helping with or making dinner compared to their parents’ reports [31]. The current study will use a dyadic approach to explore how preadolescents and parents perceive food-related autonomy and competence.

**Figure 1:**
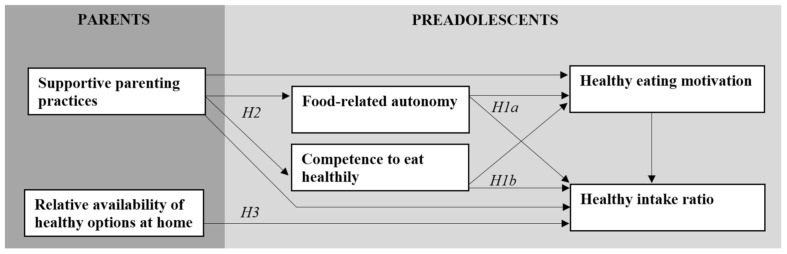
Conceptual model with hypotheses 1a (H1a), 1b (H1b), 2 (H2) and 3 (H3).

The main aim of the present study was to examine how food-related autonomy, competence and parenting practices are associated with preadolescents’ motivation to eat healthily and their actual healthy food consumption at school; see Figure 1. This will include exploring the effect of the relative availability of healthy foods at home on preadolescents’ healthy intake at school. More specifically, the following research questions will be explored:

*RQ1:* How are food-related autonomy and competence to eat healthily perceived by preadolescents and parents?
*RQ2:* How can parents support preadolescents’ food-related autonomy and competence, and what are the direct and indirect effects on their motivation to eat healthily and their healthy food consumption at school?
*RQ3:* How does the relative availability of healthy foods at home influence preadolescents’ food consumption at school?

### 1.1. Theoretical Background and Hypotheses

#### 1.1.1. Food-Related Autonomy and Competence to Make Healthy Food Choices

Adolescents have an increased desire for autonomy—the capability to think, feel and act independently [32]. This need for autonomy also applies to adolescents’ eating behavior, as adolescents value the ability to make their own food choices [33,34]. Food-related autonomy develops mainly within the home environment and includes a co-construction between parents and adolescents [35]. In general, food-related autonomy is seen as the ability to relatively independently decide which foods to choose for consumption [36].

Autonomy is a central concept of the Self-Determination Theory (SDT), a framework for explaining motivation and behavior. Particularly, autonomy plays a central role in order to feel motivated and engage in behaviors [13]. As such, adolescents with a high sense of food-related autonomy may be more likely to make healthy food choices.

Previous research using the SDT framework to explain adolescents’ eating behaviors has been mainly cross-sectional and focused on autonomous motivation, which refers to being motivated because of personal interest or value [37]. In line with SDT, autonomous motivation for healthy eating has been associated with higher intake of healthy foods among adolescents in cross-sectional studies [38,39,40,41,42] and a longitudinal study [43]. In addition, a recent study found that adolescents had lower motivation to eat fruit and vegetables when their parents reported more directive parenting behaviors, including food decisions and rules made by parents [40]. Preadolescents may have higher motivation for healthy eating and eat healthier when they experience a high sense of food-related autonomy, in which their food choices are freely chosen rather than prescribed by their parents. However, it is currently unclear how food-related autonomy influences preadolescents’ motivation to eat healthily and impacts food choices. Although it has been suggested that autonomy is related to healthy behavior choices in adolescents according to a conceptual paper [44], a previous cross-sectional study did not find associations between autonomy and consumption of fruits, vegetables and dairy foods among adolescents [36]. A possible explanation for this non-significant association includes that autonomy was measured with a single dichotomous item assessing whether parents let adolescents make their own decisions about the foods they eat, which may provide a limited representation of this construct [45]. In the current study, the following is hypothesized:

**Hypothesis** **1a** **(H1a).** *Food-related autonomy will be positively associated with preadolescents’ motivation to eat healthily and their healthy intake ratio.*

In addition to autonomy, competence is another important psychological need according to SDT [13]. Competence refers to feeling effective in producing desired outcomes—for example, healthy eating. Based on SDT, adolescents may be more motivated to eat healthily and be more likely to make healthy food choices if they feel competent. Competence is, to a certain extent, comparable to self-efficacy, which refers to confidence in the ability to carry out behaviors under challenging circumstances [46]. Self-efficacy for healthy eating has been associated with healthy food intake among adolescents [30,47,48,49,50,51]. Although competence and self-efficacy have been used interchangeably, it has been argued that these constructs are conceptually different [52]. More specifically, in contrast to self-efficacy, perceived competence includes the feeling of intrinsic satisfaction when effectively meeting a challenge [53].

Few cross-sectional studies have examined competence for healthy eating among adolescents. For example, it has been found that perceptions of competence for a healthy diet were related to higher scores on a healthy eating scale among adolescents aged 15–18 [47]. Another study conducted with normal weight and overweight/obese adolescents aged 13–18 found that perceived competence for eating healthily was associated with lower consumption of sodas and fast food per day in the entire sample [54]. As such, these studies support the link between perceived competence and healthy food intake among older adolescents. Similarly, competence for healthy eating may also be associated with healthy food choices among preadolescents. The following is hypothesized:

**Hypothesis** **1b** **(H1b).** *Competence to eat healthily will be positively associated with preadolescents’ motivation to eat healthily and their healthy intake ratio.*

#### 1.1.2. Supportive Parenting Practices Promoting Food-Related Autonomy and Competence

In line with SDT, social contexts, such as those provided by parents, are essential for supporting adolescents’ needs and their ability to engage in healthy behaviors—for example, making healthy food choices [11,12]. Several systematic reviews show that parental influence on adolescents’ eating behaviors has been studied extensively [55,56,57]. However, little is known about how parenting practices affect food-related autonomy and competence and, in turn, preadolescents’ healthy food choices. Parents can promote the development of self-determined behaviors if they are autonomy supportive and responsive [58]. Autonomy support consists of parenting behaviors that promote independent problem solving and choice, whereas responsiveness is defined as the extent to which parents are aware of children’s feelings and the way they respond to it in a supportive manner [59,60]. Children are more open to parental socialization when their parents are autonomy supportive and responsive [61], suggesting that these fundamental general parenting practices have an important influence on adolescent behaviors. Autonomy support and responsiveness are part of an authoritative parenting style, which has been related to a healthier diet and lower Body Mass Index (BMI) of children [56]. For example, a previous longitudinal study found that maternal authoritative parenting style predicted lower BMI scores of adolescents 5 years later [62]. In addition, paternal permissive parenting (high responsiveness and low demandingness) was associated with more fruit and vegetable intake among adolescent girls [62]. As such, low parental control may be associated with healthier intake among preadolescents.

In addition to research on general parenting practices, there is empirical support that parents can also influence adolescents’ behaviors through parenting practices in a specific domain (e.g., food parenting practices) [61]. A recent conceptual paper argued that certain food parenting practices may support adolescents’ needs for autonomy and competence [27]. For example, preadolescents whose parents encourage them to eat healthily may have higher food-related autonomy and competence and make healthier food choices. In addition, making healthy foods accessible in the home environment may increase preadolescents’ autonomy and competence for healthy eating and increase their consumption of healthy foods [27,63]. Previous systematic reviews [57,64] and a recent cross-sectional study [65] found positive relations between the food parenting practices of encouragement and accessibility and healthy food consumption. This study seeks evidence on how supportive general and food-specific parenting practices are associated with preadolescents’ food-related autonomy and competence. Parents who use these supportive practices may enhance preadolescents’ feelings of autonomy and competence in making healthy food choices. The following is hypothesized:

**Hypothesis** **2** **(H2).** *Supportive parenting practices will be positively associated with preadolescents’ food-related autonomy and competence.*

#### 1.1.3. Relative Availability of Healthy Foods in the Home

The home food environment is seen as an important determinant of adolescents’ eating behavior [14]. Particularly, the availability of foods is a major component of the home environment. Parents usually determine what foods are available within their home. Previous reviews have found that food availability in the home influences adolescents’ intake [14,57]. As such, the foods and beverages available to adolescents at home remain important for their intake, despite the increased opportunities to make their own food decisions (e.g., in school settings or with friends) [66]. Most previous cross-sectional studies focused on the availability of either healthy foods or unhealthy foods, e.g., [65,67]. However, the relative availability of healthy foods may provide a more complete estimate of the healthiness of the home environment. One previous cross-sectional study found that a more healthful-to-less healthful food ratio was related to higher consumption of fruit and vegetables among adolescents, suggesting that fewer unhealthy foods but more healthful foods in the home may promote adolescents to consume more healthily [68]. Therefore, it is expected that the relative availability of healthy options at home is related to higher healthy intake among preadolescents.

**Hypothesis** **3** **(H3).** *The relative availability of healthy options at home will be positively associated with preadolescents’ healthy intake ratio outside the home.*

## 2. Materials and Methods

### 2.1. Procedures

The current study is part of a larger longitudinal study examining preadolescents’ food choices during the transition from primary to secondary school. In the longitudinal study, two waves of data were collected at a primary school (school year 2018–2019) and a secondary school (school year 2019–2020). The current study used cross-sectional data from the first wave. Preadolescents were recruited through their primary schools. Schools with a regular education program were eligible to participate in the study. Primary schools in the Netherlands were invited to participate through contacting schools in the neighborhood of Wageningen University & Research and through the personal network of the researchers (*n* = 96). Schools that did not want to participate indicated that they were too busy or they were already involved in other research projects and did not want to participate. In total, 9 public primary schools agreed to participate in the study (9.38%). Based on the postal codes of the schools, 3 schools were located in low-income areas (below the Dutch median) and 6 schools were in high-income areas (above the Dutch median) [69]. The degree of urbanization was low (<1000 addresses/km^2^) for 2 schools, moderate (1000–1500 addresses/km^2^) for 1 school and high (>1500 addresses/km^2^) for 6 schools [24,70]. All schools except one had a continuous schedule, indicating that children consumed their lunch at school. After gaining permission to participate from the schools, all preadolescents in their last year of primary school (*n* = 249) and their parents were invited to participate in the study. Information sheets and consent forms were distributed by the teacher. In total, 59.04% (147/249) of all parents gave consent for their child to participate and 61.90% (91/147) of parents agreed to participate themselves as well. Three preadolescents were excluded as they were not in their last year of primary school, indicating a remaining sample size of 144 preadolescents.

In June 2019, research assistants visited each primary school that participated in the study. During the school visits, the study was explained and preadolescents were asked to give consent. The participating preadolescents and their teachers were asked to complete a questionnaire. Preadolescents who were absent during the school visits (*n* = 6) received the questionnaire by regular mail including an envelope to return the questionnaire. A total of 142 preadolescents (98.61%) and one teacher from each primary school (*n* = 9) completed the questionnaire. Children received a small present (water balloons) for their participation.

Parents who agreed to participate received an email invitation in July 2019 to complete an online questionnaire. One parent was allowed to complete the questionnaire on paper due to Repetitive Strain Injury (RSI) complaints. Reminders to complete the questionnaire were sent by email and regular mail. Parents who completed the survey received a EUR 5 voucher for an online web shop. A total of 81 parents completed the questionnaire (89.01%).

The project proposal was approved by the Social Sciences Ethics Committee of Wageningen University & Research (09215846). In addition, the study was preregistered (https://osf.io/ugrkx (accessed on 28 April 2021)).

### 2.2. Measures Preadolescents

#### 2.2.1. Healthy Intake Ratio

Different healthy and unhealthy snack and beverage categories were used to measure preadolescents’ consumption during school hours or after school away from home. The items were based on the Beverage and Snack Questionnaire [71] and a previous study assessing test–retest reliability with children aged 10–12 years [72]. The snack and beverage categories were classified as healthy or unhealthy based on guidelines issued by the Dutch Nutrition Center [73]. Healthy snack and beverage categories included fruit, vegetables, nuts, water, tea, milk and sugar-free beverages. Unhealthy categories included energy-dense snacks (cookies, biscuits, candy, chips, salty snacks and chocolate) and sugar-sweetened beverages (regular soda, fruit juice, sports/energy drinks and flavored milk/drinkable yogurt). For each category, preadolescents indicated their frequency of consumption during the past school week (0–5 days). Consequently, a healthy intake ratio was calculated based on a previous experimental study [74]. The consumption frequencies of healthy snacks or beverages were summed and then divided by the sum of the total consumption of healthy and unhealthy snacks or beverages and multiplied by 100. As the ratios for snacks and beverages were correlated (*r* = 0.23, *p* = 0.01), the average was calculated to represent preadolescents’ relative healthy intake in general. As such, a higher ratio indicated healthier consumption of snacks and beverages within preadolescents’ total consumption.

#### 2.2.2. Food-Related Autonomy

A total of 6 items were used to measure perceptions of preadolescents’ ability to make food choices. Three items were based on a previous study [75]: “I can decide for myself when to consume snacks”, “I can decide for myself how many snacks I am allowed to consume” and “My parents tell me which snacks I am allowed to consume” (reversed coding). One item was adapted from an autonomy subscale of a previous cross-sectional study [76] and included the statement, “Sometimes I consume snacks which I did not choose myself” (reversed coding). Two additional items were included: “I pack foods and drinks to bring to school” and “Are you allowed to use pocket money to buy foods or drinks?”. A 4-point scale was used, including the following responses: never, sometimes, often or always. Cronbach’s α for the items was 0.50, with an average interitem correlation of 0.15. The limited internal consistency of the scale may be explained by the multidimensional nature of the construct of food-related autonomy. As the items reflect different indices of food-related autonomy, an aggregated mean score for the items was calculated to provide a general indicator of preadolescents’ level of food-related autonomy [77]. A higher score indicated a higher perception of preadolescents’ food-related autonomy in making their own food choices.

#### 2.2.3. Competence to Eat Healthily

An adapted version of the Perceived Competence Scale (PCS) was used to measure competence to eat healthily [78]. This scale measures the degree to which people feel confident about being able to make a change toward healthy behavior. Two specific changes in healthy eating behaviors were used to assess preadolescents’ perceived ability to engage in these changes. The first change included refraining from eating candies or snacks for 7 days and only eating fruits or vegetables as a snack. The second change included refraining from drinking soft drinks or fruit juice for 7 days and only drinking water. For each change, 4 items were used. The items from the PCS were modified to relate to these changes and included “I feel confident in my ability to do this”, “I feel capable to do this”, “I am able to maintain this” and “It would be difficult for me” (reversed coding). Preadolescents were asked to rate each item as true or false. A sum score for all 8 dichotomous items (ranging from 0 to 8) was calculated, which showed good internal consistency (α = 0.90). A higher score indicated a higher perception of preadolescents’ competence to eat healthily.

#### 2.2.4. Healthy Eating Motivation

A total of 8 items were used to measure preadolescents’ autonomous motivation to engage in healthy eating. Based on a previous longitudinal study, reasons to engage in two specific healthy eating behaviors were assessed: consuming fruit/vegetables and consuming water [43]. For each behavior, 4 items were used. The items were based on previous research and included “Because it is important to me”, “Because I like it”, “Because I want this myself” and “Because it is good for my health” [43,79]. For each item, preadolescents indicated if this statement was true or false for them. A total sum score was calculated for the 8 items (ranging from 0 to 8), indicating adequate internal consistency (α = 0.78).

#### 2.2.5. School Environment and Eating Characteristics

The questionnaire for preadolescents included some additional descriptive variables about their school environment and eating characteristics. Regarding school, preadolescents were asked about their perceived school encouragement to eat healthily. Two binary items (yes/no) were used, focusing on eating fruit/vegetables and drinking water. These items were summed to indicate the degree to which preadolescents perceived their school to encourage healthy eating.

Regarding eating characteristics, preadolescents indicated on how many days they took bread to school in the past school week (0–5 days) and on how many days they had purchased foods or drinks near school in the past school week (0–5 days).

### 2.3. Measures Parents

#### 2.3.1. Food-Related Autonomy

The same items were used to measure parental perceptions of preadolescents’ ability to make food choices, except the wording of “I” was changed into “My child”.

#### 2.3.2. Competence to Eat Healthily

The same items were used to measure parental perceptions of preadolescents’ competence to eat healthily, except the wording of “I” was changed into “My child”.

#### 2.3.3. Supportive Parenting Practices

Supportive parenting practices related to preadolescents’ food-related autonomy and competence were measured with multiple items based on previous research; see Table 1. The items included both general and food-specific parenting practices.

A 5-point scale was used, ranging from “Totally disagree” to “Totally agree”. Cronbach’s α was 0.81, indicating that the items had good internal consistency. A principal component analysis, forced to one factor, revealed factor loadings ranging from 0.38 to 0.74, exceeding the recommended criterion of 0.32 for minimum loadings [80]. Consequently, a general mean score for these items was calculated to indicate the degree to which parents were supportive of autonomy and competence.

#### 2.3.4. Relative Availability of Healthy Options at Home

Parents were asked to indicate how often different snack or beverage categories were available in their home (never, sometimes, often and always) based on previous studies [68,81]. The snack and beverage categories were similar to the categories used for preadolescents’ healthy intake ratio. More specifically, healthy categories included fruit, vegetables, nuts, tea, milk and sugar-free beverages. The category water was excluded in the parent questionnaire as it was assumed that water was always available in their home. Unhealthy categories included energy-dense snacks (cookies, biscuits, candy, chips, salty snacks and chocolate) and sugar-sweetened beverages (regular soda, fruit juice, sports/energy drinks and flavored milk/drinkable yogurt). Consequently, the relative availability of healthy options at home was calculated by summing the availability frequencies of healthy snacks or beverages, dividing by the sum of the total availability of healthy and unhealthy snacks or beverages and multiplying by 100. As the ratios for snack and beverage availability were correlated (*r* = 0.45, *p* < 0.001), the average was calculated to represent the relative availability of healthy options at home in general. Consequently, a higher ratio indicated more availability of healthy snacks and beverages within the total availability of snacks and beverages in their home.

#### 2.3.5. School Environment and Eating Characteristics

The questionnaire for parents included the same questions about perceived school encouragement to eat healthily as in the questionnaire for preadolescents. Parents were also asked to indicate food purchasing behaviors of preadolescents. As the parent questionnaire was distributed in a different week, a general question about food purchasing was used (“Does your child buy foods or drinks near his/her school?”) and response options were changed into a 4-point scale from “never” to “always”. Finally, parents were asked about preadolescents’ perceived weight (too low, a bit too low, healthy weight, a bit too high or too high) and their food responsiveness using the Child Eating Behavior Questionnaire (CEBQ) subscale of food responsiveness (5 items, α = 0.82) [82].

**Table 1:** Supportive parenting practices for food-related autonomy and competence.

Parenting Practice	Items	Source
Autonomy support	1: I encourage my child to be curious, to explore, and to question things	Autonomy support subscale—Comprehensive General Parenting Questionnaire [83]
	2: I trust my child	
	3: I respect my child’s opinion and encourage him/her to express it	
	4: I encourage my child to be true to himself/herself	
	5: I encourage my child to express his/her opinions even when I do not agree with him/her	
Responsiveness	6: I know exactly when things are not going very well for my child	Responsiveness subscale—Comprehensive General Parenting Questionnaire [83]
	7: When my child is sad, I know what is going on with him/her	
	8: I feel good about the relationship I have with my child	
	9: My child and I have warm affectionate moments together	
	10: I know exactly when my child has difficulty with something	
Encouraging healthy food intake	11: I encourage my child to drink water ^a^	Comprehensive Snack Parenting Questionnaire [84], adapted by focusing on specific healthy eating behaviors
	12: I encourage my child to eat fruits and vegetables ^b^	
Making healthy foods accessible	13: I make sure my child has easy access to healthy foods ^c^	Comprehensive Snack Parenting Questionnaire [84]

Note. Items were scored on a 5-point scale ranging from “Totally disagree” to “Totally agree”. ^a^ Similar to the original item, examples were given: “For example, by encouraging my child to drink enough water, or by being positive about water.” ^b^ Similar to the original item, examples were given: “For example, by encouraging my child to eat enough vegetables during the meal, by being positive about fruits or vegetables, or by encouraging my child to eat a variety of fruits and vegetables.” ^c^ Similar to the original item, examples were given: “For example, by storing healthy foods in a place that is easily accessible to their child, or by having healthy foods, such as fruit, available in ready-to-eat form.”

### 2.4. Data Analysis

Descriptive statistics (e.g., mean, standard deviation (SD) or percentages) and Pearson’s correlation coefficients were obtained for study variables. Intraclass correlation coefficients (ICCs) were calculated to assess inter-rater agreement between preadolescents’ and parents’ reports on food-related autonomy and competence. A two-way random effects model with a single measure and absolute agreement was used for the ICCs [85]. Based on recommended guidelines, the interpretation of ICCs was based on their 95% confidence intervals, including poor agreement (<0.5), fair agreement (0.50–0.75), good agreement (0.75–0.90) or excellent agreement (>0.90) [86,87]. In addition, paired sample t-tests were used to assess differences between the mean scores for preadolescent and parent ratings on food-related autonomy and competence. To test the hypothesized model, path analysis was conducted using maximum likelihood estimation. Path analysis is an extension of multiple regression and allows for complicated models with simultaneous estimation of parameters [88]. First, the model fit was evaluated by using multiple fit indices: χ^2^, comparative fit index (CFI) and root mean square error of approximation (RMSEA). More specifically, the criteria for an acceptable model fit included a non-significant *p*-value of χ^2^, a CFI value greater than 0.90 and RMSEA ≤ 0.08 [89,90]. Second, the significance of the path coefficients was examined. Statistical analyses were performed using SPSS version 25 and AMOS version 25. The level of significance was set at *p* < 0.05.

## 3. Results

### 3.1. Participants

A total of 142 preadolescents and 81 parents (either mother or father) participated at the first wave (see Table 2). Preadolescents whose parents participated showed similar characteristics as preadolescents whose parents did not participate. For example, preadolescents’ age (*t*(140) = 0.39, *p* = 0.70), gender (χ^2^(1) = 2.84, *p* = 0.09), number of siblings (*t*(140) = 0.36, *p* = 0.72) and amount of pocket money (*t*(127) = −0.31, *p* = 0.76) were similar. Preadolescents’ ages ranged from 11 to 13 years. The majority of preadolescents and their parents were born in the Netherlands. Mainly mothers completed the parental survey. According to the parents, most preadolescents had a healthy weight. In addition, the majority of parents who completed the survey and their partners were highly educated.

### 3.2. Descriptives

**Figure 2:**
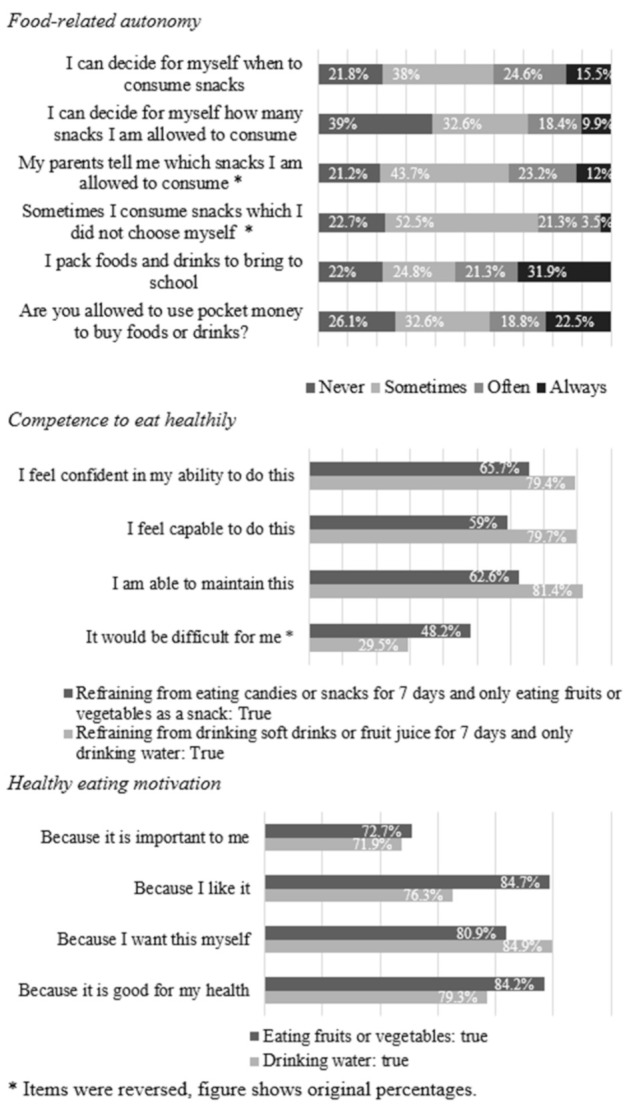
Percentage frequency distributions of items for food-related autonomy, competence to eat healthily and healthy eating motivation among preadolescents (*n* = 142).

The means and standard deviations (Table 2), correlations (Table 3) and frequency distributions of the study variables (Figure 2) are presented. On average, approximately two-thirds of the preadolescents’ total snack and beverage intake at school was healthy. Preadolescents’ healthy intake ratio was positively associated with their competence (*r* = 0.27, *p* < 0.01), motivation (*r* = 0.32, *p* < 0.01) and home availability of healthy options (*r* = 0.44, *p* < 0.01). Most preadolescents agreed to some extent that they were able to make food choices, and they perceived that they had food-related autonomy regularly (sometimes or often) (median (*Mdn)* = 1.5). Furthermore, most preadolescents agreed that they were able to make a change toward healthy eating behaviors and perceived high competence to eat healthily (*Mdn* = 6). Preadolescents were highly motivated to eat healthily (*Mdn* = 7).

On average, parents agreed with the items measuring their general and food-specific parenting practices (*Mdn* = 4.7). In addition, approximately half of the available foods and beverages at home were healthy.

### 3.3. Preadolescents’ and Parents’ Perceptions of Food-Related Autonomy and Competence

Agreement between preadolescent and parent reports on food-related autonomy and competence is shown in Table 4. The level of agreement between preadolescents and parents was poor to fair for food-related autonomy and poor for competence to eat healthily. As such, preadolescents and parents had different perceptions regarding food-related autonomy and competence. On average, preadolescents reported higher food-related autonomy than their parents would judge (*t*(80) = 9.87, *p* < 0.001). However, preadolescents perceived themselves as having lower competence to eat healthily than according to their parents’ perceptions (*t*(80) = −2.17, *p* = 0.03). Further analyses included food-related autonomy and competence ratings as assessed by preadolescents, as key outcomes (e.g., healthy eating motivation and healthy food intake) were only reported by preadolescents. Moreover, parental assessments of these constructs can be influenced by the need to create a consistent and desirable picture of the home situation.

### 3.4. Effects of Food-Related Autonomy and Competence

**Figure 3:**
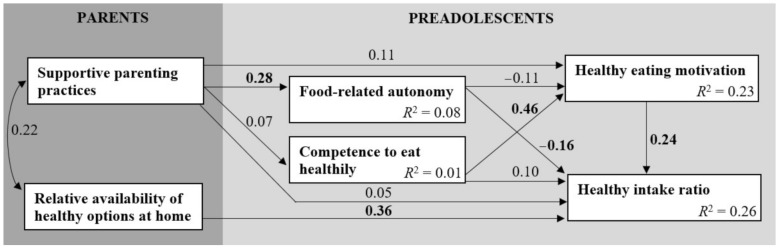
Path model with standardized coefficients (bold indicates statistically significant) and squared multiple correlations.

To test the hypothesized model, a path analysis was conducted. The model included two exogenous variables (supportive parenting practices and relative availability of healthy options at home) and the covariance between these variables, as well as four endogenous variables (food-related autonomy, competence to eat healthily, healthy eating motivation and healthy intake ratio) and error terms associated with these variables. The model (see Figure 3) had a good fit, with χ^2^ (4, *n* = 142) = 6.63, *p* = 0.16, CFI = 0.96 and RMSEA = 0.07. Unstandardized coefficients are shown in Table 5. The model accounted for 26% of the variance in preadolescents’ healthy intake ratio (Figure 3).

It was hypothesized that food-related autonomy would be positively associated with preadolescents’ motivation to eat healthily and their healthy intake ratio (H1a). As can be seen in Figure 3, food-related autonomy had no significant association with healthy eating motivation. In contrast with the hypothesis, food-related autonomy was negatively associated with preadolescents’ healthy intake ratio (β = −0.16, *p* = 0.04), indicating that preadolescents who were more able to make food choices consumed less healthy foods within their total intake.

However, H1b was confirmed. More specifically, preadolescents who felt competent to eat healthily were more motivated to eat healthily (β = 0.46, *p* < 0.001), which was positively associated with their healthy intake ratio (β = 0.24, *p* = 0.01). As the two paths between competence and motivation and motivation and healthy intake ratio were both significant, healthy eating motivation acted as a mediator [91].

### 3.5. Parenting Practices Supporting Food-Related Autonomy and Competence

It was hypothesized that supportive parenting practices would be positively associated with preadolescents’ food-related autonomy and competence (H2). As expected, the path between supportive parenting practices and food-related autonomy was significant (see Figure 3), indicating that more parental support was associated with higher food-related autonomy (β = 0.28, *p* = 0.01). However, supportive parenting practices were not associated with preadolescents’ competence to eat healthily. Therefore, H2 was only supported for food-related autonomy.

### 3.6. Effect of Relative Availability of Healthy Options at Home

As hypothesized in H3, a direct effect was found for the relative availability of healthy options at home on the healthy intake ratio of preadolescents (see Figure 3; β = 0.36, *p* < 0.001). This effect indicates that home availability of healthy options was associated with higher consumption of healthy foods as compared to preadolescents’ total intake. Therefore, H3 was confirmed.

### 3.7. School Environment and Eating Characteristics

Approximately two-thirds of preadolescents agreed that their school encouraged them to eat fruit or vegetables, whereas approximately one-third agreed that their school encouraged them to drink water (Table 2). On average, preadolescents took bread to school on nearly all days in the past school week. Preadolescents who took bread to school more often were more motivated to eat healthily (*r* = 0.20, *p* = 0.02). On the other hand, purchasing foods was infrequent in the past school week (Table 2). Food purchasing was negatively associated with preadolescents’ healthy intake ratio (*r* = −0.25, *p* < 0.01), indicating that preadolescents who bought more foods consumed less healthy foods in their total intake. In addition, food purchasing was related to more food-related autonomy (*r* = 0.22, *p* = 0.01), and less competence (*r* = −0.17, *p* = 0.05) and motivation for healthy eating (*r* = −0.20, *p* = 0.02).

Furthermore, preadolescents’ food responsiveness as reported by parents was associated with less food-related autonomy (*r* = −0.24, *p* = 0.03), showing that preadolescents who are responsive to foods were less able to make food choices.

## 4. Discussion

This cross-sectional study used the Self-Determination Theory framework to examine how food-related autonomy, competence and parenting practices are related to preadolescents’ healthy eating motivation and food consumption at school. This also included testing the effect of the relative availability of healthy foods at home on preadolescents’ food consumption outside the home. Early adolescence, at 10–14 years, is seen as a developmental transition with major changes [22]. Early adolescents gain more independence and frequently make their own food choices [6,7,8]. The present study extends previous research by focusing on this unique age group and assessing both parents’ and preadolescents’ perceptions on food-related autonomy and competence. The SDT model seems partly appropriate to explain preadolescents’ food intake at school based on the present study.

The findings of the study showed that supportive parenting practices were related to higher food-related autonomy, which was associated with less relative healthy intake at school. In contrast to expectations guided by the SDT model, preadolescents with more food-related autonomy were not more motivated to eat healthily or make healthier food choices. This corresponds with research among younger children (around 9 years old), which showed that being a permissive parent with little demands and control in food-specific situations is associated with poor diet quality [92]. However, the effects of parental control on food intake seem less clear among adolescents. For example, a longitudinal study found that more maternal control over children’s behaviors was associated with less fruit and vegetable intake, suggesting that preferences for healthy options may decrease due to parental control [93]. In contrast, a previous cross-sectional study among adolescents found that more restrictive parenting practices were associated with lower consumption of soft drinks [94]. Further research is needed to clarify the associations between food-related autonomy and food intake of adolescents.

An alternative explanation for these findings could be that preadolescents chose less healthy foods as a form of parental reactance or to conform with peers [95]. Consequently, healthy options when eating outside the home may not be appealing for preadolescents [96]. This is supported by our finding that food-related autonomy was generally low among preadolescents, as the majority of preadolescents only agreed to some extent that they were able to make food choices. In addition, preadolescents did not purchase foods often. The level of food-related autonomy may increase as preadolescents move to secondary school, where they are able to buy food themselves in the school canteen or shops near the school during lunch breaks [6,97]. However, it is unknown how this possible increase in food-related autonomy impacts the healthiness of their food choices.

Considering competence to eat healthily, the associations were in line with the SDT model. More specifically, preadolescents who felt competent to eat healthily were more motivated to eat healthily as well. In turn, more healthy eating motivation was positively associated with preadolescents’ relative healthy intake at school. These results are in line with previous cross-sectional studies on competence [47,54] and healthy eating motivation ([38,39,40,41,42] (cross-sectional), [43] (longitudinal)). The findings of the present study further support the important role of competence to eat healthily and healthy eating motivation as predictors of preadolescents’ healthy intake at school. As this study did not find significant associations between supportive parenting practices and preadolescents’ perceived competence to eat healthily, it is important to gain more insight into how parents can support this need. In the current study, supportive parenting practices were mainly focused on autonomy support. Other parenting practices specifically related to competence may have more supportive influence [27].

Furthermore, the present study found that higher relative availability of healthy options at home was related to more consumption of healthy foods within preadolescents’ total intake at school. This finding is consistent with previous systematic reviews [14,57] and underscores the need for parents to create a relatively healthy home environment in order to guide their children to choose healthy foods. Future interventions should aim to support parents with strategies to promote preadolescents’ independent healthy food choices, including moderation of unhealthy foods. This is something that parents describe as difficult, particularly when adolescents see these attempts as pressuring and forceful [98]. Imposing overly restrictive and controlling rules can have an opposite effect by making unhealthy foods look like a “forbidden fruit” and, in this way, increases desire and intake. The literature on intuitive and internally regulated eating shows that a relaxed relationship with indulgent foods is positively associated with food intake regulation and weight maintenance [99]. In facing the external world, preadolescents are learning to make their own decisions. Making healthy foods available at home may set a norm of what is acceptable eating behavior, and, in this way, it may impact eating behavior outside the home. Healthy eating practices at home have been shown to influence food choices by shaping adolescents’ feelings about healthy eating [26].

Preadolescents reported higher food-related autonomy than their parents would judge. Apparently, parents think that they have more control over their preadolescents’ snack choices than adolescents experience themselves. Moreover, preadolescents perceived themselves as having lower competence to eat healthily than according to their parents’ perceptions. It could be that they lack confidence, but it may also be related to parents’ tendency to overestimate their child’s diet quality [100]. In general, it seems that parents perceive their own food-related behaviors as more positive than adolescents do and vice versa. For example, low agreement between adolescent and parent reports has been found for perceptions about food parenting practices, e.g., [28,29], indicating that parents report more frequent use of healthy food parenting practices than adolescents do. Parent reports have been found to be higher than adolescent reports for various food parenting practices, including food availability and accessibility [29,30,101], meal routines [31,102], modeling [101] and food rules [29]. According to a meta-analysis [103] and a systematic review [104], parents are prone to biases in perceptions of their child, such as a lack of awareness of overweight in their own children. If parents do not properly understand what their child is ready to handle in terms of competences and ability to make independent decisions, this may have implications for how well parents are able to recognize that changes are necessary. To measure competence and autonomy, it is important that researchers are aware of differences in perceptions and take children’s measures as leading in predicting their eating behavior.

Despite the strengths of the study, including the multi-informant dyadic data of preadolescents and parents and the measurement of supportive parenting focused on both general and food-specific practices [105], there are some limitations that need to be acknowledged. First, self-report methods were used, which may have impacted the accuracy of preadolescents’ food intake measurement at school [106] and may have led to potential social desirability bias among the preadolescents and parents. However, the provision of socially desirable answers does not seem to have happened when parents estimated their child’s weight status, as the reported proportion of overweight children is consistent with recent Dutch figures on overweight among children [107]. Second, the measures of food-related autonomy, competence and motivation were not validated prior to use, thus limiting the interpretability of the findings. This applies in particular to food-related autonomy, as this measure had limited internal consistency. However, the measures were founded on previous cross-sectional studies and adjusted to the age group and specific healthy eating behaviors to increase construct validity. Third, the sample of the study was relatively small, which may have impacted the statistical power. In addition, many parents who participated in the study were highly educated, which limits the generalizability of the findings.

Future research could consider using the SDT model to explain preadolescents’ eating behaviors. Additional studies are needed to confirm the findings from this study and gain more insight into the role of food-related autonomy, competence and parental support. Expanded definitions and measurements of the constructs relevant to preadolescents should be used. In addition, future longitudinal studies could examine the causal relationships between SDT constructs and preadolescents’ eating behaviors. As parenting practices were only related to food-related autonomy, future studies could investigate the role of other parenting practices that may support competence for healthy eating. Consequently, the results could inform evidence-based guidelines for parents on how they can help their children to develop a sense of competence in making independent healthy food choices. Moreover, as peers become more important during adolescence and have been found to negatively impact healthy eating behavior [108], it would be relevant to explore how the need of relatedness as proposed in SDT is associated with healthy eating motivation and healthy food consumption. Furthermore, considering the positive impact of competence to eat healthily on preadolescents’ healthy eating motivation and intake, it would be interesting to translate these findings into a theory-based intervention to promote healthy eating and explore the impact on actual food intake.

## 5. Conclusions

In conclusion, early adolescence is a time in which the dynamics of the relationship between parents and their children is changing. Moreover, there are more snacking occasions unsupervised by an adult in environments with a plentiful supply of tempting unhealthy foods and increased social influence of peers. This study provides support for the use of the SDT model to explain preadolescents’ healthy food intake at school. In particular, competence to eat healthily and healthy eating motivation are important predictors of preadolescents’ healthy intake. In addition, parents can promote healthy intake by creating a home environment with a higher relative availability of healthy food options. The findings from the study advance the understanding of individual and environmental factors guiding preadolescents to make healthy food choices on their own.

## Figures and Tables

**Table 2:** Descriptive statistics of study variables.

Variable	Preadolescents (*n* = 142)	Preadolescent–Parent Dyads (*n* = 81)	
	*M* (*SD*)	Preadolescents *M* (*SD*)	Parents *M* (*SD*)
Preadolescents			
Age (years)	12.18 (0.43)	12.17 (0.38)	
Gender			
Boys	55.6%	61.7%	
Girls	44.4%	38.3%	
Ethnicity: Dutch	97.9%	98.8%	
Siblings	1.49 (0.91)	1.47 (0.92)	
0 siblings	6.3%	8.6%	
1 sibling	53.5%	49.4%	
2 or more siblings	40.2%	41.9%	
Pocket money per week (EUR)	2.44 (2.23)	2.49 (2.44)	
Perceived weight status (parent-reported)			
Underweight		18.5%	
Healthy weight		64.2%	
Overweight		17.3%	
Healthy intake ratio	62.91 (24.98)	63.82 (25.23)	
Food-related autonomy ^a^	1.51 (0.53)	1.54 (0.57)	0.99 (0.35)
Competence to eat healthily ^b^	5.41 (2.79)	5.37 (2.87)	6.17 (1.82)
Healthy eating motivation	6.19 (2.08)	6.12 (1.99)	
Number of schooldays bread is taken to school	4.48 (1.08)	4.46 (1.14)	
Food purchasing	0.44 (0.91)	0.46 (0.90)	0.49 (0.59)
Food responsiveness (parent-reported)		2.24 (0.67)	
School encouragement to eat FV: Yes	62.4%	59.7%	79%
School encouragement to drink water: Yes	36.2%	40.7%	60.5%
Parents			
Age			43.04 (5.35)
Relationship to child			
Mother			91.4%
Father			8.6%
Ethnicity of mother: Dutch	90.8%		95.1%
Ethnicity of father: Dutch	88.7%		96.3%
Relationship: Yes			84%
Highest educational level			
Low			1.2%
Middle			34.6%
High			64.2%
Highest educational level of partner			
Low			2.9%
Middle			39.7%
High			57.4%
Difficulties with financial situation: No			95.1%
Supportive parenting practices			4.59 (0.34)
Relative availability of healthy options at home			49.16 (5.68)

Note. ^a^ rated on a 0–3 scale. Care should be taken to interpret this scale, as Cronbach’s alpha is 0.50; ^b^ rated on a 0–8 scale. Abbreviations: *n* = number of participants, *M* = mean, SD = standard deviation.

**Table 3:** Pearson’s correlation coefficients between study variables.

Variable	1	2	3	4	5	6	7	8	9	10
1: Healthy intake ratio										
2: Food-related autonomy	−0.16									
3: Competence to eat healthily	0.27 **	0.04								
4: Healthy eating motivation	0.32 **	−0.06	0.46 **							
5: Supportive parenting practices (parent-reported)	0.12	0.31 **	0.05	0.08						
6: Relative availability of healthy options at home (parent-reported)	0.44 **	−0.02	0.21	0.05	0.23 *					
7: School encouragement to eat healthily	0.13	−0.06	−0.16	−0.03	−0.08	−0.11				
8: Number of schooldays bread is taken to school	0.16	−0.12	0.17	0.20 *	−0.12	0.19	−0.10			
9: Food purchasing	−0.25 **	0.22 **	−0.17 *	−0.20 *	−0.05	−0.09	−0.01	−0.05		
10: Food responsiveness (parent-reported)	0.03	−0.24 *	0.02	0.01	−0.07	−0.10	0.01	0.05	−0.12	

Note. * *p* < 0.05, ** *p* < 0.01.

**Table 4:** Agreement between preadolescent and parent reports (*n* = 81) on food-related autonomy and competence.

Variable	Preadolescents *M* (*SD*)	Parents *M* (*SD*)	Mean Difference (95% CI)	ICC (95% CI)
Food-related autonomy (mean score total scale 0–3)	1.54 (0.57)	0.99 (0.35)	0.55 (0.44–0.66) *	0.27 (−0.07–0.54)
I can decide for myself when to consume snacks	1.35 (0.99)	0.94 (0.64)		0.09 (−0.10–0.29)
I can decide for myself how many snacks I am allowed to consume	1.04 (1.07)	0.50 (0.60)		0.09 (−0.09–0.28)
My parents tell me which snacks I am allowed to consume ^a^	1.77 (0.98)	1.00 (0.76)		0.11 (−0.07–0.29)
Sometimes I consume snacks which I did not choose myself ^a^	1.89 (0.81)	1.75 (0.52)		0.26 (0.05–0.46)
I pack foods and drinks to bring to school	1.71 (1.12)	1.08 (1.09)		0.44 (0.17–0.63)
Are you allowed to use pocket money to buy foods or drinks?	1.45 (1.23)	0.66 (0.64)		0.19 (−0.02–0.39)
Competence to eat healthily ^b^(sum score total scale 0–8)	5.37 (2.87)	6.17 (1.82)	−0.80 (−1.54–−0.07) *	0.04 (−0.17–0.24)

Note. ^a^ Reversed coding. ^b^ Only sum score is shown due to the dichotomous items. * *p* < 0.05. Abbreviations: *M* = mean, SD = standard deviation, CI = confidence interval, ICC = intraclass correlation coefficient.

**Table 5:** Unstandardized parameters of the path model.

Effect	Estimate	*SE*	*t*	*p*
Supportive parenting practices → Food-related autonomy	0.44	0.16	2.66	0.01
Supportive parenting practices → Competence to eat healthily	0.58	0.90	0.64	0.52
Food-related autonomy → Healthy eating motivation	−0.43	0.31	−1.37	0.17
Competence to eat healthily → Healthy eating motivation	0.34	0.06	6.18	<0.001
Supportive parenting practices → Healthy eating motivation	0.66	0.63	1.05	0.29
Healthy eating motivation → Healthy intake ratio	2.85	1.03	2.78	0.01
Relative availability of healthy options at home → Healthy intake ratio	1.56	0.41	3.80	<0.001
Food-related autonomy → Healthy intake ratio	−7.68	3.80	−2.02	0.04
Competence to eat healthily → Healthy intake ratio	0.90	0.75	1.20	0.23
Supportive parenting practices → Healthy intake ratio	3.99	7.65	0.52	0.60

## Data Availability

The data that support the central findings of this study will be made available on https://zenodo.org/ (accessed on 28 April 2021) by the authors.
